# Repartitioning brain glucose: serine–nucleotide dependency sensitizes glioblastoma therapy

**DOI:** 10.1038/s41392-025-02506-2

**Published:** 2025-12-19

**Authors:** Yanggang Hong, Chunyan Hua, Min Wu

**Affiliations:** 1 The Second School of Medicine, Wenzhou Medical University, Wenzhou, Zhejiang China; 2 School of Basic Medical Sciences, Wenzhou Medical University, Wenzhou, Zhejiang China; 3 RNA Research and Drug Discovery Center, Wenzhou Institute, University of Chinese Academy of Sciences, Wenzhou, Zhejiang China

**Keywords:** Molecular neuroscience, Cancer metabolism

**Fig. 1:**
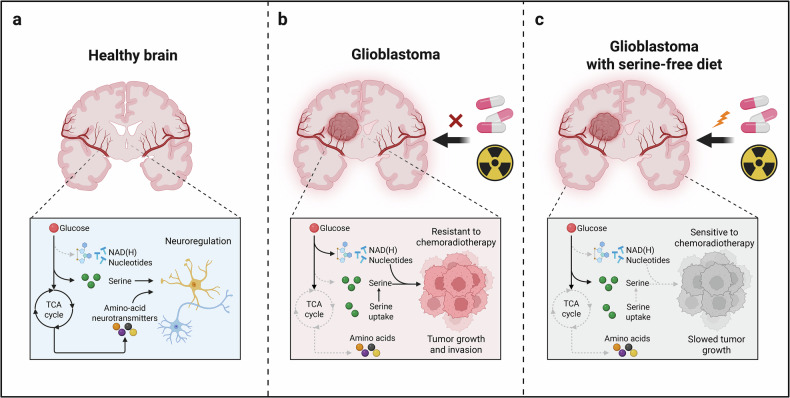
Glucose fate in the cortex versus glioblastoma and the effect of dietary serine restriction. **a** Healthy brain: Glucose is preferentially oxidized through the TCA cycle and supports the synthesis of amino-acid neurotransmitters; serine is largely supplied by de novo synthesis for neuroregulation. **b** Glioblastoma: Despite comparable glucose uptake, tumors downshift the TCA/neurotransmitter routes and reallocate glucose carbon to nucleotide and NAD(H) biosynthesis while increasing serine uptake, sustaining growth and reducing sensitivity to chemoradiotherapy. **c** Glioblastoma with a serine-free diet: Limiting exogenous serine drives glucose back toward serine synthesis, lowers the nucleotide/NAD(H) supply, and sensitizes tumors to chemoradiotherapy, slowing growth. Pills and radiation icons denote standard treatment; red cross indicates poor response under baseline conditions; lightning indicates enhanced response with serine restriction. TCA, tricarboxylic acid; NAD(H), NAD^+^/NADH; “serine-free diet” denotes serine/glycine restriction (−SG). The figure was created with BioRender.com

A recent study published in *Nature* by Scott et al.^1^ uses in vivo isotope tracing in patients and orthotopic mouse models to show that whereas the cortex channels glucose into oxidative/tricarboxylic acid (TCA) and neurotransmitter metabolism, glioblastoma (GBM) repartitions glucose carbon toward nucleotide and NAD(H) biosynthesis. Leveraging the preference of GBM for imported serine, dietary serine/glycine restriction reroutes carbon, depresses nucleotide production, and sensitizes tumors to chemoradiation (Fig. 1).

As the most aggressive primary brain tumor, GBM has a median survival of less than 15 months despite maximal safe resection and standard Stupp chemoradiation, underscoring the need for therapies that exploit tumor–brain metabolic differences.^2,3^ Scott et al. reframe the classic “GBM is highly glycolytic” narrative from how much glucose tumors take up to what they do with it. Despite similar glucose entry into tumors and the cortex, GBM suppresses glucose oxidation/TCA-linked neurotransmitters and reallocates glucose carbons toward nucleotides and NAD^+^/NADH production—the biosynthetic currency of proliferation and treatment resistance. Functionally, GBM strongly relies on environmental serine; restricting serine/glycine forces glucose back into serine synthesis, depletes nucleotide labeling, and improves therapeutic responses in selected models.

Brain health depends on glucose not only as fuel but also as a carbon source for neurotransmitters; indiscriminate glycolysis blockade therefore risks cortical toxicity. Fluorine-18 fluorodeoxyglucose positron emission tomography has already shown that the cortex and GBM both import glucose robustly, yet clinical efforts have struggled to translate “glucose addiction” into durable benefit.^4^ The key gap has been the lack of in vivo fate mapping to resolve how glucose-derived carbons traverse the intact brain and tumor across therapy-relevant timescales. A strategy that identifies downstream liabilities (e.g., nucleotides, one-carbon/serine metabolism) rather than glucose entry itself could offer selectivity without sacrificing neuronal function.

Scott et al. infused [U^13^C]glucose into patients intraoperatively and into intracranial patient-derived xenografts, capturing the cortex and enhancing and non-enhancing tumors for liquid chromatography‒mass spectrometry (LC‒MS) and spatial matrix-assisted laser desorption/ionization (MALDI) MS to map labeling patterns. They built an ordinary differential-equation-based metabolic-flux analysis (MFA) model to convert time-course isotopologues into absolute fluxes and extended it into a dynamic, non-steady-state framework to quantify rapid pathway responses after radiation. Pathway-specific validation used ^15^N-amide-glutamine (de novo nucleotides) and ^15^N_4_-inosine (purine salvage), and serine handling was probed by direct ^13^C_3_-serine infusion to compare uptake in the tumor versus cortex.

GBM rewires glucose away from physiological conditions toward biosynthesis. The cortex exhibited higher glucose-derived labeling of TCA intermediates and neurotransmitters (glutamate, GABA, aspartate, and glutamine), whereas GBM showed the opposite pattern‒reduced oxidative/TCA-cycle and neurotransmitter labeling, corroborated spatially by MALDI and by downregulation of neurotransmission transcripts. In contrast, GBM displayed elevated labeling of purines, pyrimidines, and NAD^+^/NADH, with a notable bias toward the GMP arm. MFA converted enrichment into a mechanism: de novo flux from IMP to GMP was increased in GBM, and uridine salvage dominated pyrimidine synthesis in both tissues, whereas de novo UMP synthesis was greater in GBM. Tracer validations with ^15^N glutamine and ^15^N_4_ inosine aligned with the model, confirming that tumors truly accelerate nucleotide production, not just label uptake.

When therapy acutely increases purine flux, serine is a lever. Within approximately one hour of cranial radiation, GBM transiently upregulated de novo IMP synthesis and channeled it into GMP while reducing AMP, which was consistent with the demand for DNA repair, whereas the cortex was largely unchanged. ^15^N tracers confirmed the flux shift, representing the first intracranial measurement of post-RT purine synthesis dynamics in GBM. This purine surge highlights the focus on serine, a key precursor for one-carbon and nucleotide biosynthesis: GBM prioritized environmental serine (higher ^13^C_3_-serine uptake than did the cortex across models), whereas the cortex predominantly made serine de novo from glucose.^5^

In addition to nucleotide synthesis, the metabolic switch from the pentose phosphate pathway (PPP) to the serine synthesis pathway (SSP) under serine/glycine deficiency raises broader biosynthetic questions. Both pathways generate NADPH and nucleotides from glucose, yet SSP uniquely supplies one-carbon units, amino acids, and S-adenosylmethionine, linking serine metabolism to redox balance, methylation and protein synthesis. This suggests that GBM response to serine restriction may reflect integrated demands for amino acid and methyl-donor homeostasis rather than nucleotide scarcity alone. Future studies should determine whether serine deprivation induces transcriptional or post-translational regulation of key enzymes within PPP and SSP and how oncogenic and tumor-suppressor mutations modulate these responses, which could help distinguish responders from non-responders to serine/glycine-restricted diets.

Serine/glycine restriction re-routes carbon and sensitizes GBM. Removing serine/glycine from media or the diet decreased the level of intracellular serine, pushed the remaining serine to be glucose-derived, and broadly reduced the nucleotide level in GBM. In vivo, a serine/glycine-free (−SG) diet decreased the amount of circulating serine and remodeled tumors but not cortical metabolomes; tumors with high serine-uptake/low-synthesis signatures shrank, and survival improved, whereas a model in which serine was synthesized from glucose had a minimal response. Crucially, −SG enhanced the efficacy of standard chemoradiation across models. Direct in vivo tracing under −SG confirmed the mechanism: GBM increased m + 3 serine from [U^13^C]glucose and showed reduced glucose-derived nucleotide labeling, exactly the carbon-repartitioning predicted.

Serine handling is heterogeneous and shaped by the tumor microenvironment. Many GBMs favor uptake over synthesis, but not all, and serine availability is influenced by non-malignant cells, such as astrocytes, stromal cells, microglia and tumor-associated macrophages, which may upregulate SSP and export serine or one-carbon units to tumor cells. Such intercellular metabolic crosstalk could buffer the impact of systemic serine/glycine restriction on intratumoral serine pools. Dissecting how oncogenic genotypes and microenvironmental cell types jointly determine serine sourcing will be critical for understanding why some tumors respond to serine/glycine-restricted interventions, whereas others maintain serine-supported nucleotide synthesis.

Taken together, by mapping glucose fate in vivo, Scott et al. reveal a serine–nucleotide dependency in GBM that can be targeted without globally blocking glucose uptake. Their work argues for biomarker-guided serine/glycine restriction, potentially synchronized with genotoxic therapy, to cap DNA-repair capacity and sensitize tumors while sparing cortical metabolism.
